# Engineered recombinant bacterial collagen as an alternative collagen-based biomaterial for tissue engineering

**DOI:** 10.3389/fchem.2014.00040

**Published:** 2014-06-23

**Authors:** Bo An, David L. Kaplan, Barbara Brodsky

**Affiliations:** Department of Biomedical Engineering, Tufts UniversityMedford, MA, USA

**Keywords:** collagen, triple helix, recombinant expression, biomaterials, tissue engineering

The key structural and signaling roles of collagen in the extracellular matrix (ECM) make it an attractive biomaterial for tissue engineering, but there are limitations in the standardization and purity of natural collagen sources currently available for such applications (Ruggiero and Koch, 2008; Werkmeister and Ramshaw, 2012). Significant effort has been made to produce human collagen in recombinant systems, such as yeast, insect cells and plants (Ruggiero et al., 2000; Myllyharju, 2009). However, the requirement for post-translational proline hydroxylation has proven to be a significant obstacle in achieving large scale production. Recent findings of collagen-like proteins in bacteria suggest these may represent alternative biosynthetic collagen materials which may complement current sources.

Over the past 10 years, collagen-like proteins have been identified from numerous bacterial genomes database based on the signature (Gly-Xaa-Yaa)_n_ repeating amino acid sequence characteristic of the collagen triple-helix (Rasmussen et al., 2003; Yu et al., 2014). Some of these collagen-like molecules may function as virulence factors by bacteria to evade the immune system of higher animals or to interact with surface receptors or with other ECM molecules necessary to promote host cell invasion (Humtsoe et al., 2005). More than 100 putative collagen-like proteins have been identified in bacterial genomes, of which eight have been recombinantly expressed in *Escherichia coli* (see Yu et al., 2014 for review). All eight expressed bacterial collagens were shown to form stable triple-helices with T_m~35–39°C_. *E. coli* and most bacteria lack prolyl hydroxylase, so this high stability is attained in the absence of hydroxyproline (Hyp), a post-translationally modified amino acid known to be critical to the thermal stability of mammalian collagens. Initial interest in bacterial collagen-like proteins focused on their roles in pathogenesis. However, recent work has focused on one specific bacterial collagen protein, designated Scl2, to demonstrate the utility of recombinant bacterial collagen as a tool for defining collagen sequence/structure/function relationships and for establishing a class of novel collagen-based biomaterials.

The gram positive bacterium *Streptococcus pyogenes* contains two collagen-like proteins, Scl1 and Scl2, which have been well characterized in terms of structure and functional properties (Lukomski et al., 2001; Xu et al., 2002; Mohs et al., 2007; Caswell et al., 2008). The Scl2 protein includes an N-terminal globular trimerization domain adjacent to a (Gly-Xaa-Yaa)_79_ core collagen-like domain. It has been possible to generate constructs in a recombinant *E. coli* system with various sequence modifications of Scl2 and to establish large scale production methods. Based on recent progress, we suggest that the Scl2 recombinant bacterial collagen system has advantages compared to recombinant human collagen strategies for large scale production and biomedical applications, and may serve as a prototype for engineering novel collagen-based biomaterials.

## Stable triple helical protein without hydroxyproline

The recombinant Scl2 protein and its modified variants are able to form a triple helix with stability similar to that of mammalian collagens (T_m_~37°C) even though there is no Pro hydroxylation. In animal collagens, Pro residues in the Y positions of the repeating Gly-X-Y sequence are hydroxylated to Hyp, and this hydroxylation is required to stabilize the triple-helix: T_m_ of hydroxylated collagen = 37°C, while T_m_ of unhydroxylated collagen = 26°C (Berg and Prockop, 1973; Jimenez et al., 1973). The bacterial collagen appears to compensate for the absence of Hyp with electrostatic interactions (Mohs et al., 2007). In yeast, plants, and other expression systems for human collagens, the genes for the alpha and beta subunits of human prolyl hydroxylase (P4H) must be introduced to form stable collagen molecules (Ruggiero et al., 2000; Myllyharju, 2009; Xu et al., 2011; Shoseyov et al., 2014). However, P4H activity and hydroxylation levels are highly dependent on expression conditions, such as the gene copy ratio of collagen to hydroxylase, concentration of cofactors, induction time, and sequence (Chan et al., 2012), so generating optimal hydroxylation to model native human collagen has been challenging in systems with inserted P4H genes and even in mammalian cell expression systems. The bacterial collagen-like proteins are highly compatible with expression in *E. coli*, and the lack of requirement for any post-translational modifications presents the potential for producing large quantities of recombinant stable triple-helical proteins for biomaterials applications.

## Mix and match functional modular units

**Figure 1:**
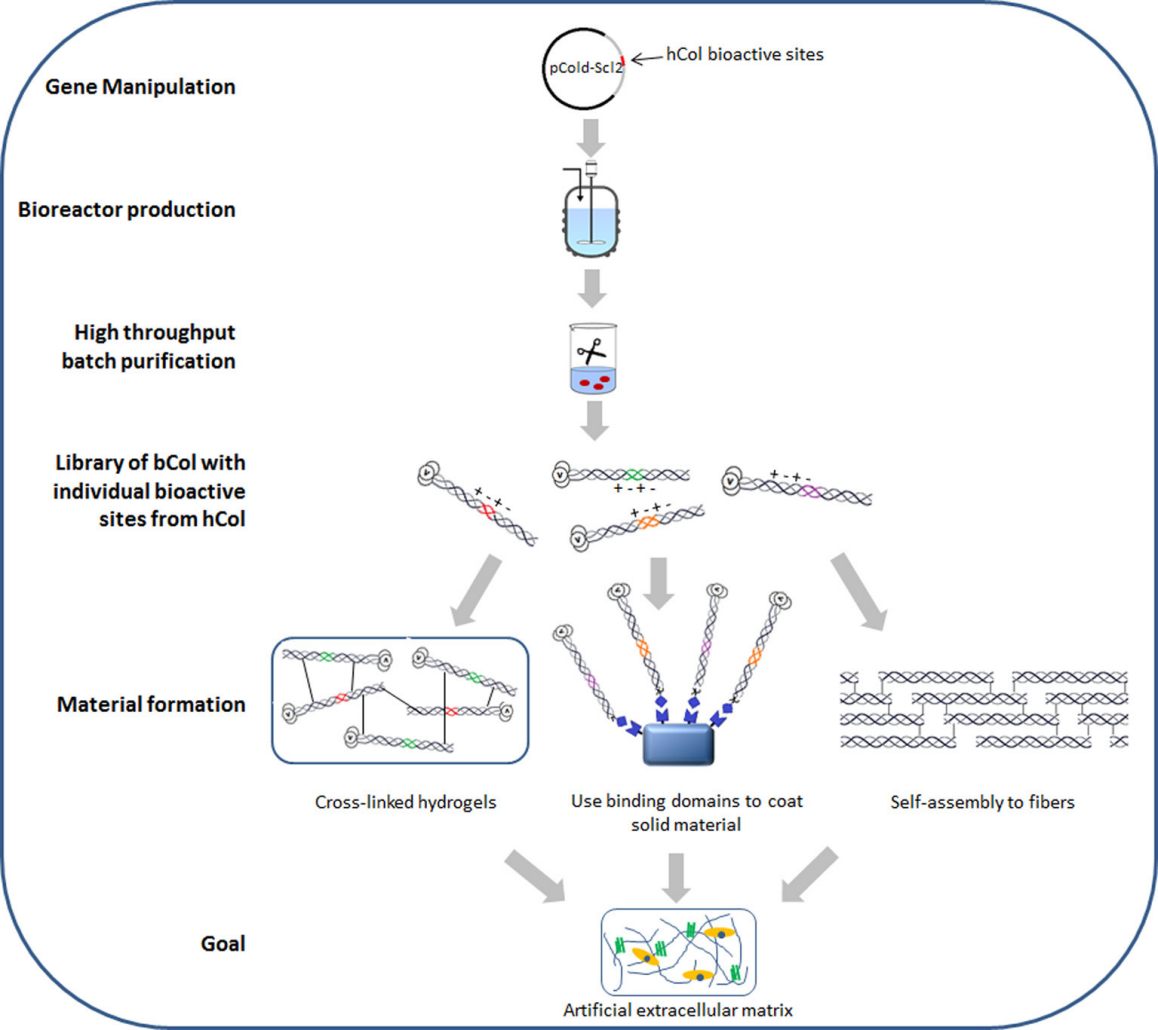
**Schematic diagram of recombinant bacterial collagen production method and material fabrication strategies**. hCol, human collagen; bCol, recombinant bacterial collagen Scl2.

In addition to production advantages, the ability to express the collagen-like protein in *E. coli* allows easy manipulation of the sequence to enhance biomaterial properties. Insertion of known human collagen binding sites within Scl2 presents the possibility of designing collagen-like materials with defined biological properties. The native human collagen sequence contains more than 40 binding sites which interact with other biologically functional molecules, including cell receptors, other ECM proteins and collagenases (MMPs) (Kadler, 1994). Examination of the Scl2 recombinant protein suggests that this particular bacterial collagen-like protein lacks any known biologically active sites, making it a convenient blank slate for the introduction of human collagen ligand binding sites (Caswell et al., 2008; Cosgriff-Hernandez et al., 2010). Various human-derived collagen short amino acid sequences (2–6 triplets) with identified bioactive sites have been inserted into the Scl2 collagen-like domain, including sites for binding integrin α2β 1 (Seo et al., 2010), fibronectin (An et al., 2014), heparin (Peng et al., 2013), and MMPs (Yu et al., 2012). The inserted sequences have conferred the expected biological activities on the Scl2 protein based on *in vitro* binding, cleavage, and cell culture assays. The Scl2 sequence will apparently fold the short human collagen insert into a triple helix, while the correctly folded human collagen sequence imparts the corresponding biological function to the chimeric protein. The introduction of the integrin and fibronectin binding sites promoted the growth of different types of mammalian cells *in vitro*, while Scl2 molecules with MMP cleavage sites show enzyme cleavage at the native position. More collagen bioactive sites are being identified each year primarily through synthetic collagen mimetic Toolkit peptides (Farndale et al., 2008), providing an increasing pool of candidates for a recombinant chimeric bacteria-human library with human collagen functions. Such a recombinant library is particularly useful for applying a synthetic biology “plug and play” concept to future biomaterial designs (Figure 1). Only those collagen biological functions that are required for a particular application will be selected and incorporated into a final product, leading to precise tuning for each application.

## Molecular and higher order structure

With recombinant DNA technologies, it has been possible to manipulate the Scl2 protein, studying individual fragments of the triple-helix (Yu et al., 2011) or duplicating the entire triple-helix portion (Yoshizumi et al., 2009), while maintaining secondary structure and stability. Scl2 is a homotrimer, serving as a model for homotrimeric collagens such as type II collagen in cartilage. To better mimic heterotrimeric collagens such as the dominant type I collagen in bone and tendon or type IV collagen in basement membrane, it may be possible to replace the natural trimerization V domain with a heterotrimic coiled coil (Nautiyal et al., 1995) or an electrostatic interaction chain selection mechanism (Jalan et al., 2013).

While facile manipulation of the Scl2 protein can optimize molecular and biological properties, successful biomaterial applications will require optimization of material properties as well. The most abundant animal collagens self-associate into characteristic periodic cross-linked fibrils, which provide the mechanical properties for tissues and biomaterials. Thus far, Scl2 exhibits limited ability to form fibrillar structures (Yoshizumi et al., 2009). The Scl2 triple-helix is only about 1/5 of the length of the human fibrillar collagen triple-helix, and it is possible that increasing its length will promote self-association. The *E. coli* system usually has an upper threshold for the size of the recombinant protein it can produce, but it may be possible to transfer the Scl2 expression system into yeast or insect cells to further increase its chain length. Crystal structures on small collagen model peptides and studies on recombinant tobacco type I collagen suggest Hyp may be essential for collagen fibril formation (Kramer et al., 2000; Perret et al., 2001). Thus, it may be necessary to introduce P4H genes into the bacterial system in order to induce fibril formation, even though the Hyp is not necessary to form the stable triple-helix. Alternative approaches to attaining an optimal self-supporting material from Scl2 include chemical modifications such as glutaraldehyde vapor or 1-ethyl-3-(3-dimethylaminopropyl)carbodiimide or crosslinking by poly(ethylene glycol) diacrylate to form interchain networks (Cosgriff-Hernandez et al., 2010; Peng et al., 2010). Chemical modifications are simple and effective, but the level of crosslinking maybe difficult to control, especially in large 3D scaffolds, and a high degree of cross-linking may limit accessibility to biologically active sites within the collagen material. Another strategy recently reported involved non-covalent binding of Scl2 constructs with fibronectin and integrin interaction sites to solid silk protein material, generating porous silk scaffolds with improved support of cell growth (An et al., 2013) (Figure 1).

## Purification, scalability, and projected cost

Animal extracted collagens are produced in large quantity and are generally inexpensive. However, difficulties in developing standardized preparations of these collagens and in producing minor types of collagens that are free of collagen I and biological contaminants are major limitations. Potential infectious and allergic risks of animal collagen products are also a concern. The recombinant bacterial collagens have attached tags, such as His-tag and Strep II tags to simplify standardized chromatographic purification. A high-throughput batch purification methodology for Scl2 has also been developed (Peng et al., 2014). Native triple helical sequences are resistant to digestion by non-specific enzymes such as trypsin and chymotrypsin, and trypsin treatment of acidified cell lysate resulted in purified triple helical bacterial collagen. Enzymatic digestion during the purification process ensures the final product will be free of non-triple helical contaminants, which is important for quality control for industrial production. Recombinant expression of proteins in *E. coli* is a mature industrial process with excellent scalability. This has already been demonstrated for Scl2 production (Peng et al., 2012) with an average yield of 0.2–0.3 g/L of purified collagen protein in traditional shaking flask culture and up to 9.5 g/L in high density fed-batch culture. With the ease of generating different Scl2 constructs through molecular cloning, the overall resource cost for producing this highly tunable bacterial collagen material will likely be lower than recombinant collagen obtained from mammalian cell or even transgenic systems.

## Toward *in vivo* applications

Translational science remains a less explored area for recombinant collagens. Recombinant human collagens obtained from systems with high scalability, such as *Pichia* and *Nicotiana*, have been formulated into hydrogels and explored for their potential as artificial cornea (Merrett et al., 2008) and wound dressings (Shilo et al., 2013), respectively. In terms of bacterial collagen, Scl2 has been shown to be non-immunogenic, non-cytotoxic and non-thrombogenic (Peng et al., 2010). Recently, poly(ethylene glycol) crosslinked bioactive Scl2 hydrogels have been reinforced with an electrospun polyurethane mesh to achieve suitable biomechanical property for vascular grafts (Browning et al., 2012). The rate of cell migration is tunable through altering protein concentration in the material. Further investigation, especially the degradation and turnover time of Scl2 material *in vivo* is needed to evaluate its suitability as medical implants.

## Critical properties for a successful collagen production strategy

Figure 1 illustrates the current production method and material fabrication strategies for recombinant bacterial collagen Scl2. Large amounts of bioactive collagen molecules can be produced with simple gene manipulation, large scale production and high throughput purification. Functional collagen proteins could then be cross-linked into hydrogels or used as coating on other solid materials. Conditions and sequence manipulations which would trigger Scl2 self-assembly into large fibers are currently under investigation. Such fabricated materials could lead to the ultimate goal of designing and developing artificial extracellular matrices, an objective important for tissue engineering as well as biomedical fields. With its high tunability and scalability accompanied by low complexity and cost, we believe the Scl2 recombinant bacterial collagen system has clear advantages which could not only circumvent the difficulties seen for recombinant human collagens, but also open up a brand new pathway for collagen production.

## Long term prospects

Extracted bovine collagen is likely to remain a staple for biomaterial construction due to its low cost and useful material properties, but challenges in standardization and concerns for bioburdens, as well as a desire to modify collagen sequence and function, will lead to continued interest in research and applications with recombinant collagens. As described here for the specific Scl2 protein from *S pyogenes*, bacterial collagens represent a biosynthetic ground up approach, where a triple-helical non-animal collagen molecule with no specific bioactivity can be designed to include desired interactions and regulated degradation. Although research has focused on Scl2, the approach could be extended to other collagen-like proteins from bacteria and viruses, which may bring new properties and variations useful for biomaterials. Fundamental studies on Scl2 and other bacterial collagens will continue to elucidate basic features of collagen, with a focus on chemistry, sequence, biological activity, and self-assembly. The success of the bacterial collagen approach toward biomaterial applications will depend on developing the capacity to generate biomaterials with desirable biomechanical properties useful for *in vivo* applications.

### Conflict of interest statement

The authors declare that the research was conducted in the absence of any commercial or financial relationships that could be construed as a potential conflict of interest.
